# Supplementary material for: Plant Regeneration via Organogenesis in Jerusalem Artichokes and Comparative Analysis of Endogenous Hormones and Antioxidant Enzymes in Typical and Atypical Shoots
Source: Plants (Basel). 2023 Nov 7;12(22):3789. doi: 10.3390/plants12223789 (PMC10675715; doi:10.3390/plants12223789)
Supplement: Supplementary file 1 [file plants-12-03789-s001.zip › Supplemental Figure.pdf]

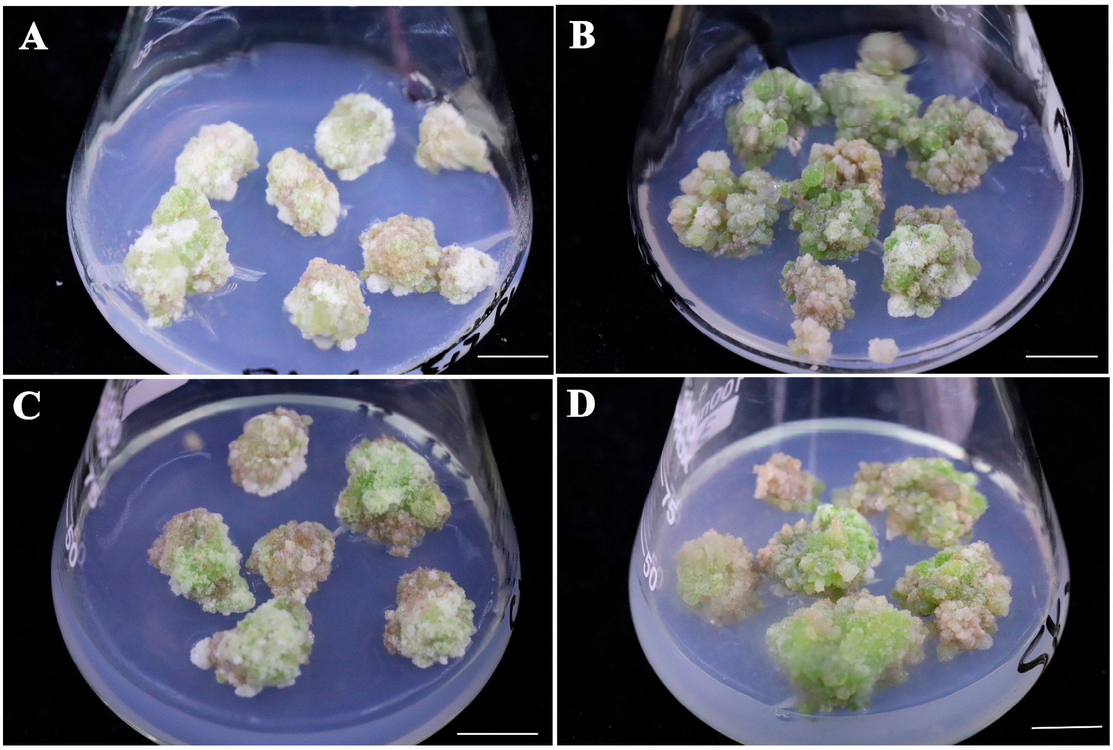

Figure S1. The stem-derived callus of Jerusalem artichoke cultured on MS basal media supplemented with 0.5 mg/L BA and 0.3 mg/L NAA **(A)**, 0.5 mg/L BA and 0.6 mg/L NAA **(B),** 1.0 mg/L BA and 0.3 mg/L NAA **(C)** , and 1.0 mg/L BA and 0.6 mg/L NAA **(D)** after 60 days culture. Bars = 1 cm.
