# Supplementary material for: Plant Regeneration via Organogenesis in Jerusalem Artichokes and Comparative Analysis of Endogenous Hormones and Antioxidant Enzymes in Typical and Atypical Shoots
Source: Plants (Basel). 2023 Nov 7;12(22):3789. doi: 10.3390/plants12223789 (PMC10675715; doi:10.3390/plants12223789)
Supplement: Supplementary file 1 [file plants-12-03789-s001.zip › Supplemental Table.pdf]

Supplemental Table

Table S1. List of primer sequences used for SSR analysis in this study. These primers were designed by Yang et al. (2018) based on the transcriptome sequences of Jerusalem artichoke (*Helianthus tuberosus*)

Marker name	Motif	No. of repeats	Forward primer	Reverse primer	Annealing temperature (℃)
JS4	AT	6	ACATATCCACCACCAACCGT	CCACTTCGCCAGTGGTTAGT	57.0
JS5	GAT	6	CTGCTGCAGATGATGATGGT	GATCCGATACAGGAAGCCAA	54.7
JS6	GTG	5	GGTGTGTGACTTGGTGGTTG	CTGCACACTGCACACCTTTT	56.6
JS7	TGG	5	ATTTCTTCATCATACGGCGG	CTACTTCATCCACCATCGCC	54.1
JS8	GCAG	7	GGGGCCAAAGTGTAATTTCC	GTTCGCATTTGACAATCCCT	53.6
JS10	CCA	6	TCAATCAACCCACACACACC	AGAAGGTGTTCCAGTCACGC	56.6
JS11	GT	7	TGAAATTGTGGTGGGGTTTT	CCCCCATAAACCCTAGCTTC	53.8
JS13	CT	6	TTTACTGGTGGCTCCGTTTC	GGAGTGGAGAGGAGGAGGAG	57.3
JS18	CAC	7	AATTGGCGGAGTGTCAAGAG	TAAGGATGCACGCTGACTTG	55.2
JS19	AGAT	4	GGGCCACTTTTCTCTGAACA	TTAATGGTGGAATCAACCCAA	53.2
JS20	CCA	8	GAAGTTGGTTGCTTTTTCGG	TTTTCCCAATCAAACTCAACTG	51.5
